# A New Method of Closing Large Defects in the Intestines

**Published:** 1891-09

**Authors:** 


A NEW METHOD OF CLOSING LARGE DEFECTS IN THE INTESTINES.—In a paper read before the French Congress for Surgery, March 30, 1891, Dr. Chaput, of Paris, stated that he had closed intestinal perforations in dogs, by stitching to the margin of the openings pieces of iodoform gauze folded in five or six layers. These remained several weeks *in situ* and were finally discharged into the intestinal canal. Owing to the fact that the omentum adheres immediately to the Gauze, a thick, protective layer is formed which not only closes the defect, but also preserves the peritoneum from infection.

The iodoform gauze, therefore, acts as a temporary plug, enabling the omentum to organize and permanently occlude the opening. In consequence of the structure of the guaze and the anti-septic properties of iodoform, an emigration of bacteria from the intestine to the peritoneal cavity is prevented. In three of the dogs experimented upon complete healing was obtained The author thinks that this method may prove serviceable in cases of gunshot wounds of the intestines, and for the intestinal perforations produced during laparotomies. It might also be useful in the majority of the operations upon the intestines.—*Bulletin Medical.*—*Southern Clinic.*

